# Supplementary material for: Biomimetic computer-to-brain communication enhancing naturalistic touch sensations via peripheral nerve stimulation
Source: Nat Commun. 2024 Feb 20;15:1151. doi: 10.1038/s41467-024-45190-6 (PMC10879152; doi:10.1038/s41467-024-45190-6)
Supplement: Supplementary file 1 — Supplementary Information [file 41467_2024_45190_MOESM1_ESM.pdf]

SUPPLEMENTARY MATERIALS

Biomimetic computer-to-brain communication enhancing naturalistic touch sensations via peripheral nerve stimulation

Giacomo Valle[1,†], Natalija Katic Secerovic[2,1,3,†], Dominic Eggemann[1], Oleg Gorskii[4,5,6], Natalia Pavlova[4], Francesco M. Petrini[7], Paul Cvancara[8], Thomas Stieglitz[8], Pavel Musienko[4,9,10], Marko Bumbasirevic[11] and Stanisa Raspopovic[1,*]

[1] Laboratory for Neuroengineering, Department of Health Sciences and Technology, Institute for Robotics and Intelligent Systems, ETH Zürich, Zürich, Switzerland.

[2] School of Electrical Engineering, University of Belgrade, 11000 Belgrade, Serbia.

[3] The Mihajlo Pupin Institute, University of Belgrade, 11000 Belgrade, Serbia

[4] Laboratory for Neuroprosthetics, Institute of Translational Biomedicine, Saint-Petersburg State University, Saint-Petersburg, Russia.

[5] Laboratory for Neuromodulation, Pavlov Institute of Physiology, Russian Academy of Sciences, Saint Petersburg, 199034, Russia.

[6] Center for Biomedical Engineering, National University of Science and Technology "MISIS", 119049 Moscow, Russia

[7] SensArs Neuroprosthetics, Saint-Sulpice, CH-1025, Switzerland

[8] Laboratory for Biomedical Microtechnology, Department of Microsystems Engineering–IMTEK, Bernstein Center, BrainLinks-BrainTools Center of Excellence, University of Freiburg, D-79110 Freiburg, Germany.

[9] Sirius University of Science and Technology, Neuroscience Program, Sirius, Russia

[10] Laboratory for Neurorehabilitation Technologies, Life Improvement by Future Technologies Center "LIFT", Moscow, Russia

[11] Orthopaedic Surgery Department, School of Medicine, University of Belgrade, 11000 Belgrade, Serbia.

*Corresponding author: stanisa.raspopovic@hest.ethz.ch

† Equal contribution

List of Supplementary Materials:

Fig. S1. Synchronization of the neural activity evoked in the spinal cord.

Fig. S2. Biomimetic neurostimulation elicits more natural sensations than non-biomimetic approaches in Participant 3.

Fig. S3. Electrically-evoked sensation naturalness related to different projected fields location.

Fig. S4. Walking speed baseline in the Cognitive Dual Task (CDT).

Fig. S5. No learning effect on spelling performance.

Table S1. Participants' demographics.

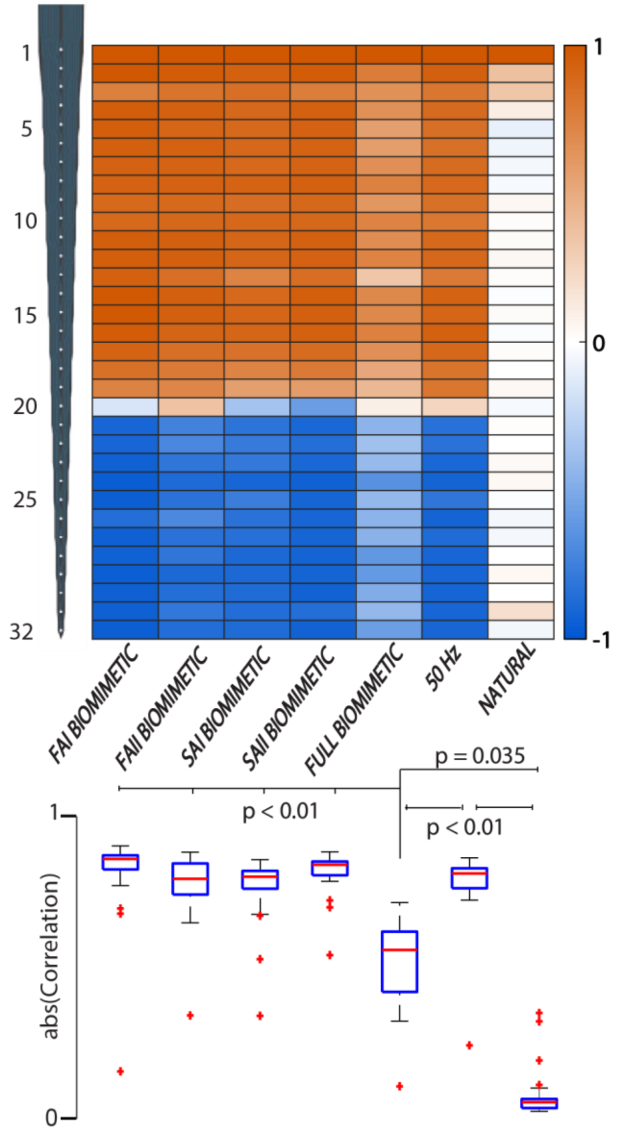

Fig S1. Synchronization of the neural activity evoked in the spinal cord. We compared and presented the correlation between the neural local field potential recording in the first electrode channel with all the other channel recordings. Upper part: Correlation values are color coded and presented along the spinal array electrode axes. Bottom part: We compared absolute values of correlation coefficient of natural with every biomimetic condition as well as 50 Hz stimulation with FULL biomimetic and natural touch condition with Kruskal-Wallis test. Boxplots: The central mark indicates the median, and the bottom and top edges of the box indicate the 25th and 75th percentiles, respectively. The whiskers extend to the most extreme data points not considered outliers, and the outliers are plotted individually using the red '+' symbol. (p values: FA1-FULL biom: <0.001; FA2- FULL biom: 0.001; SA1- FULL biom: 0.01; SA2- FULL biom: <0.001; 50Hz-FULL biom: 0.001; Natural- FULL biom: 0.035; 50Hz-Natural: p<0.001; significance level 1%, chi-square value 187.4). Source data are provided as a Source Data file.

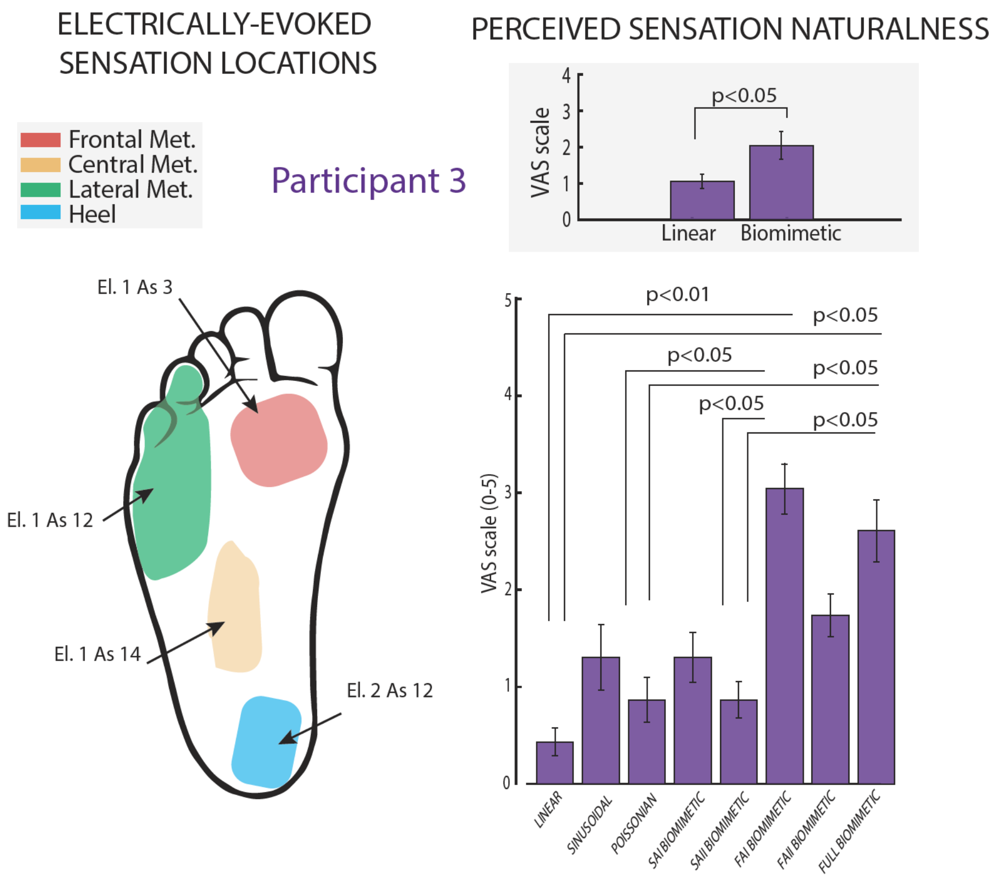

Fig S2. Biomimetic neurostimulation elicits more natural sensations than non-biomimetic approaches in Participant 3. Projective fields map of Participant 3 related to the active sites adopted to electrically stimulate the tibial nerve. Different colors show the 4 main regions of the phantom foot (Frontal, Lateral and Central Metatarsus, and Heel). Naturalness ratings (VAS scale 0-5) of the perceived sensation elicited exploiting different stimulation strategies. Insets: Group comparison between linear vs biomimetic stimulations. We compared the conditions using Kruskal-Wallis test (n=16 stimulation repetitions). Post-hoc correction was executed. BIOM-LIN: p=0.0026, f=1.01. Data are presented as mean values +/- standard deviation. Source data are provided as a Source Data file.

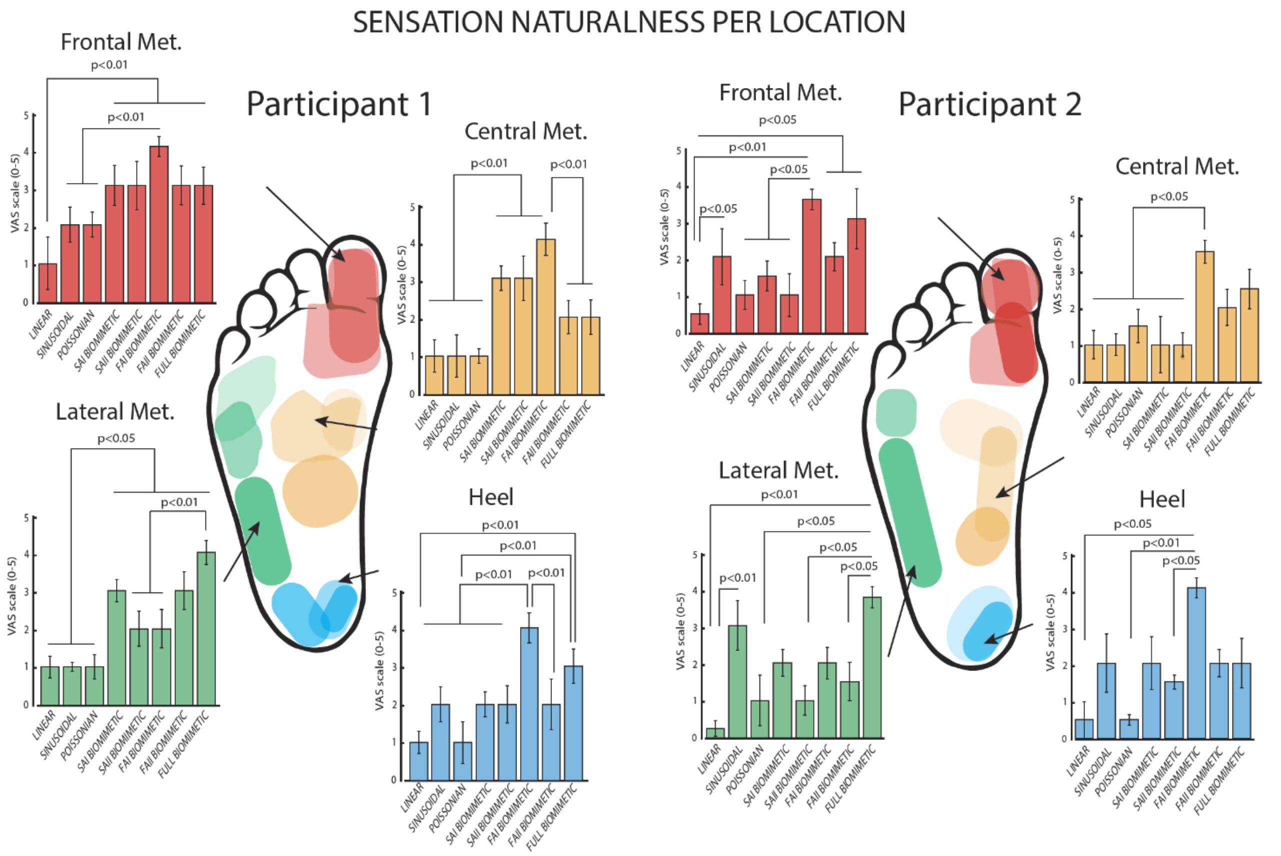

Fig S3. Electrically-evoked sensation naturalness related to different projected fields location. Projective field maps of two implanted participants (1 & 2) related to the active sites adopted to electrically stimulate the nerves. Different colors show the 4 main regions of the phantom foot (Frontal, Lateral and Central Metatarsus, and Heel). Naturalness ratings (VAS scale 0-5) of the perceived sensations breakdown per location of the projected fields. We compared the conditions using Kruskal-Wallis test (participant 1: frontal met: n=15; central met.: n=15; lateral met: n=21; heel: n= 24; participant 2: frontal met: n=6; central met.: n=9; lateral met: n=9; heel: n=9 per stimulation repetition per condition). Post-hoc correction was executed. BIOM-LIN: p=0.0026, f=1.01. Data are presented as median values +/- standard deviation. Source data are provided as a Source Data file.

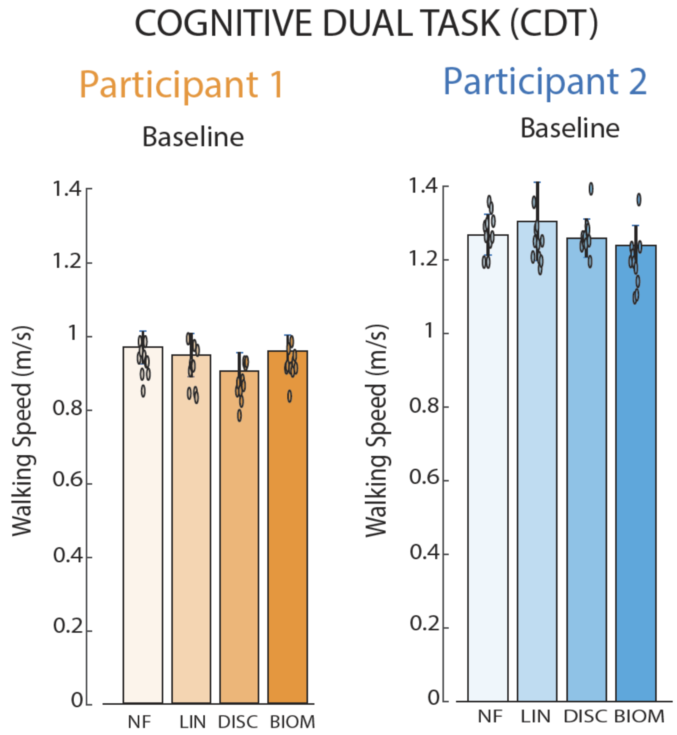

Fig S4. Walking speed baseline in the Cognitive Dual Task (CDT). Motor performance (Walking Speed – m/s) of Participant 1 & 2 in the Cognitive Dual Task (CDT) at baseline (no mental task). The tested conditions are NF (No Feedback), LIN (Linear Neurostimulation), DISC (Discrete Neurostimulation) and BIOM (Biomimetic Neurostimulation), n=10 for each condition, for both participants. The ellipses overlapped with bar plot represent single data points. Data are presented as median values +/- standard deviation. Source data are provided as a Source Data file.

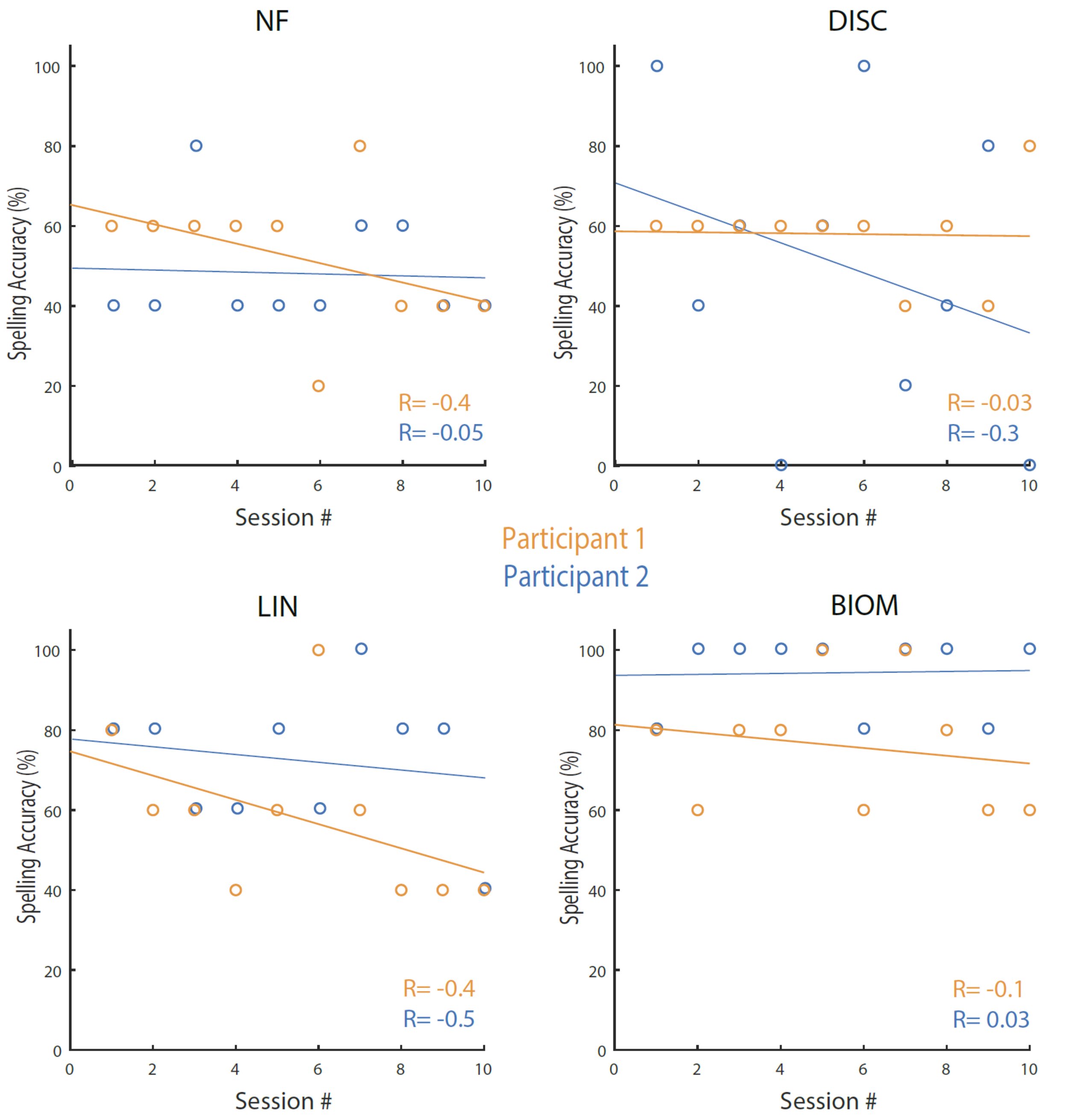

Fig S5. No learning effect on spelling performance. Spearman's rank correlation coefficients on the spelling accuracy across sessions for participant 1 & 2. The tested conditions are NF (No Feedback), LIN (Linear Neurostimulation), DISC (Discrete Neurostimulation) and BIOM (Biomimetic Neurostimulation). Source data are provided as a Source Data file.

Table S1. Participants' demographics.

Patient	Cause of Amputation	Level and Side of Amputation	Amputation Time	Phantom Limb Pain	Own Prosthesis	Frequency of Use
S1	Trauma	Distal two-thirds of right thigh	2	Medium	Passive prosthesis (3R80-Ottobock)	Daily
S2	Trauma	Distal two-thirds of right thigh	12	Medium	Passive prosthesis (3R80-Ottobock)	Daily
S3	Trauma	Distal two-thirds of left thigh	7	Low	Passive prosthesis (3R80-Ottobock)	Daily

The original protocol is written in Serbian language. This is its translation in English.

This supplement contains the following items:

1. Original protocol.
2. Original statistical analysis plan, final statistical analysis plan.

1.	PROTOCOL	PAGE:
	Original Protocol V1 1 Sep 2016	2-18
2.	STATISTICAL ANALYSIS	
	Original statistical analysis plan	19-20

The original protocol is written in Serbian language. This is its translation in English.

1. PROTOCOL

Study Name:
Restoring feelings from a lost limb to amputated people to avoid falls, healthy walking, pain-relief and prosthesis embodiment (Feel-Again)

Acronym of the Study:
Feel-Again

Main researcher:
Aleksandar Lešić, Francesco Petrini

Clinics where they will be conducted:
Clinic for Orthopedic Surgery and Traumatology: Department of Reconstructive Surgery and Microsurgery, Clinical Center of Serbia, Belgrade.
Special Hospital for Rehabilitation and Ophthalmic Prosthetics, Belgrade.

Included research groups:
Swiss Institute of Technology Lausanne, Switzerland
IMTEK, Albert-Ludwigs University, Freiburg, Germany
University & Axonic, Montpellier - LIRMM, France - LIRMM, France
SensArs Neuroprosthetics, Lausanne, Switzerland
OSSSUR, Reykjavik, Iceland

Sponsor: Prof. Aleksandar Lešić (address: Koste Todorovića 26, 11000 Belgrade).

Reference number: FeelAgain 1.1.

Protocol Version 1.1.

Date 1.09.2016

The original protocol is written in Serbian language. This is its translation in English.

Protocol Synopsis

People with amputation of the legs suffer from a complete lack of sensations from the available commercial prostheses. This excludes the central nervous system from correct sensorimotor integration, and causes serious problems such as: i) asymmetric walking and balance (greater reliance on healthy legs), causing bone pathologies (arthritis and osteoporosis) and higher metabolic consumption (with frequent cardiac problems), ii) falls due to unexpected obstacles or holes, iii) feeling of a prosthesis as a foreign body, resulting in the abandonment of the prosthesis, and iv) neuropathic phantom pain.

The aim of the research is to test an innovative bionic prosthesis in volunteers with leg amputation, with the hypothesis that the restoration of the sensations from the amputated leg will enable them to avoid or reduce the above complications.
The prosthesis consists of: i) intraneural electrodes (TIME electrodes produced by IMTEK, Germany), which will be implanted in the rest of the sciatic nerve, ii) neurostimulators (external neurostimulator from the University of Montpellier and AXONIC, France), which will inject a current into the nervous system via electrodes, iii) a controller of a system associated with iv) a sensorized insole that is placed under the prosthetic foot (a sensorized insole manufactured by the Swiss Institute of Technology, Lausanne and SensArs, Switzerland) iv) commercial prostheses (produced by OSSSUR -a, Iceland).

The principle of work consists in the translation, through algorithms implemented in the system controller, of the pressure signal that senses the foot in the electrical stimulation parameters of the remaining nerve. In this way, the patient will feel direct and natural, the foot sensations during the walk, and, therefore, be able to correct his/her motor controls, resulting in a proper walk. Innovative electrical stimulation of the remaining sciatic nerve, via intraneural electrodes, will cause natural feelings from the foot and the leg, which play a key role in maintaining balance and in correct walking.

Clinical examination, which contains a detailed set of tests, including ethical and psychological aspects, will explore the effect of restoring feelings on: preventing falls, walking and balance dynamics, metabolic costs, phantom pain, and embodiment (the degree of acceptance of the prosthesis as part of the body). Previously, through the same technology, the work of the same institutions involved in this project enabled the restitution of the hand in subjects with amputation of the upper extremities. They could feel the touch of the missing hands, and use them in a bidirecfional prosthesis control. Their phantom pain has been reduced, and the embodiment of the prosthesis has increased.

"Feel Again" aims to translate these effects into persons with leg amputation and to achieve therapeutic effects that would act against the health problems described.

This study is launched at the researchers' initiative. The data will not be used for certification of future medical products (such as CE certificate). The working title of this clinical study is: "Feel-Again".

The original protocol is written in Serbian language. This is its translation in English.

1. INTRODUCTION

Proposal abstract

The clinical investigation described here aims at evaluating (in selected transfemoral amputees) a lower limb prosthesis providing sensory feedback. The device, called SENSY, is constituted by i) sciatic nerve stimulating system constituted by implantable intraneural electrodes from IMTEK and external neural stimulator from Grapevine/AXONIC, iii) sensorized sole from SensArs to apply under the prosthetic foot and driving the stimulating system. The device can be used by amputees equipped with a lower limb prosthesis. The sensorized sole is designed to be put under the prosthetic foot in a shoe. During this clinical investigation, SENSY will be used together with the CE-marked prosthesis from OSSUR.
Lower limb amputees suffer complete **lack of sensory feedback** of current available prostheses, which excludes central nervous system from the correct sensory-motor integration. It causes serious problems as: **falls** due to unexpected perturbations, **asymmetric walking and balance** inducing bone pathologies, higher **power** consumption (with occasional hearth failures), feeling the prosthesis as a **foreign body** (with consequent abandonment) and **phantom limb pain** occurrence.
Clinical testing with detailed battery of tests, including also ethical and psychological aspects, will explore fall prevention, walking dynamics, metabolic cost, phantom limb pain level, embodiment, providing the objective assessment of SENSY.
The clinical trial is an investigator-initiated clinical trial. Data will not be used for medical device approval (CE mark).
We call this clinical evaluation FeelAgain.

Historical background and bio-medical translational research

There are 5 million amputees, only in USA and Europe and 475000 new amputations are performed every year (www.amputee-coalition.org). 80% of these subjects lost the lower limb. Leg amputees **lack sensory feedback** and have **limited voluntary control** of currently available prostheses[1]. These limitations do not allow correct generation of postural reflexes at the spinal level and overall correct sensory-motor integration between the user's central nervous system and the artificial limbs.
Users experience dangerous **falls**[2], do not manage to maintain symmetry during standing and walking[3-4], i.e. they tend to shift more weight and to have a prolonged stance phase on the sound limb than on the prosthetic limb[5-7], because of the lack of sensory feedback and no controllability of the prosthesis itself. Resulting **abnormal kinematics and postural asymmetries** can, after long-term use of the prosthesis, lead to **musculoskeletal diseases** as knee and hip osteoarthritis, osteoporosis, and back pain[8-9]. Moreover, since they exert unnatural compensatory movements with prosthetic and healthy leg and body, they face **augmented metabolic cost**, then fatigue and occasionally **hearth failures**[10-11]. As such, an amputee, especially a thigh-level ones (transfemoral (TF), is faced with several challenges in daily life situations. Sitting and standing up, running, shuffling and carrying loads can be a difficult and even dangerous task for a TF amputee. Moreover, 50-80% of amputees report neuropathic pain from the missing extremity, which is called **phantom limb pain (PLP)**[12] and for whom an effective treatment is not available[13]. Finally, the users do **not perceive the prosthesis as a part of their own body**[14], which increases the cognitive effort when using the device itself[15], affecting its acceptability (low embodiment)[16-17] and causing a reduction in the confidence of the subject in its use (i.e. they are afraid to fall if relying over it) resulting in 60% of lower limb amputees abandoning the prosthesis (i.e. they do not use it and do not walk anymore)[18-19].
Currently amputees completely lack sensory feedback from their prostheses. Sensory feedback provided by foot sole mechanoreceptors is important for controlling balance and movement in

humans[20-24]. Lower-limb amputees rely on very unnatural control and practically inexistent and often uncomfortable haptic feedback from the stump-socket interaction to monitor ground contact, counteract interaction with obstacles, stabilize balance and walk symmetrically. Therefore they have very poor perception of the instantaneous behaviour or position or motion of the prosthetic device, which in facts does not allow them to operate in closed loop control. Many, if not most, of the drawbacks associated with operating the device arise from the lack of proper sensory feedback of the lost limb, which then do not allow to link intent, residual limb motion and resulting device behaviour. Partial or full restoration of the afferent information path would allow closing that gap, which currently stands wide open. Therefore, the development of a leg prosthesis with sensory feedback is an unmet need.

Concept

Recent/ongoing research projects (e.g., EU projects: CYBERHAND, TIME, EPIONE, NEBIAS) have shown how implantable peripheral neural interfaces can significantly improve the usability of hand prostheses. In particular, the new transversal intraneural interface[25] developed in the framework of the TIME EU project showed to provide very promising results[26, 27]. Advanced leg mechatronic solutions are arising from projects such as CYBERLEG even if with reduced actuation and sensorization. Moreover, there is complete lack of naturally controllable prostheses due to the limits of current interfaces both as devices (selectivity, stability), and their policy of use (decoding and encoding), which would offer physiological channels for efferent control and afferent perception. The control of walking and balance is achieved by a complex combination of descending motor commands (going from the central and peripheral nervous systems, CNS and PNS respectively, to the muscles) and ascending sensory (proprioceptive, tactile, etc.) information. This hierarchical and highly flexible structure is learnt during childhood[28] and can, to some extent, be re-learnt after e.g. stroke and amputation. Starting from the previous scientific rationales, mainly in upper-limb, FeelAgain has developed the first lower-limb prosthesis with sensory feedback. The aim of FeelAgain is to test such device on transfemoral lower limb amputees to assess the efficacy in enabling physiological gait to amputees, increasing the embodiment of the prosthesis and reducing phantom pain.
The FeelAgain “bionic” leg prosthesis will be based on the following building blocks (Figure 1):

1. Leg-prosthesis with actuated knee and ankle, to create a range of natural and effective walking options.
2. Intraneural transversal electrode able to create a stable, intimate, and effective connection with the afferent fibers of sciatic nerve for sensory feedback.
3. Sensorized sole ported with biomimetic model-based, encoding algorithm to “translate” the information recorded by the sensors, during walking and standing, into stimulation patters to be delivered to the afferent nerves;
4. External stimulator to generate the electrical stimuli and provide a pathway from the sole to the implanted electrodes;

The effectiveness of the FeelAgain will be verified with 3 transfemoral amputees.

The original protocol is written in Serbian language. This is its translation in English.

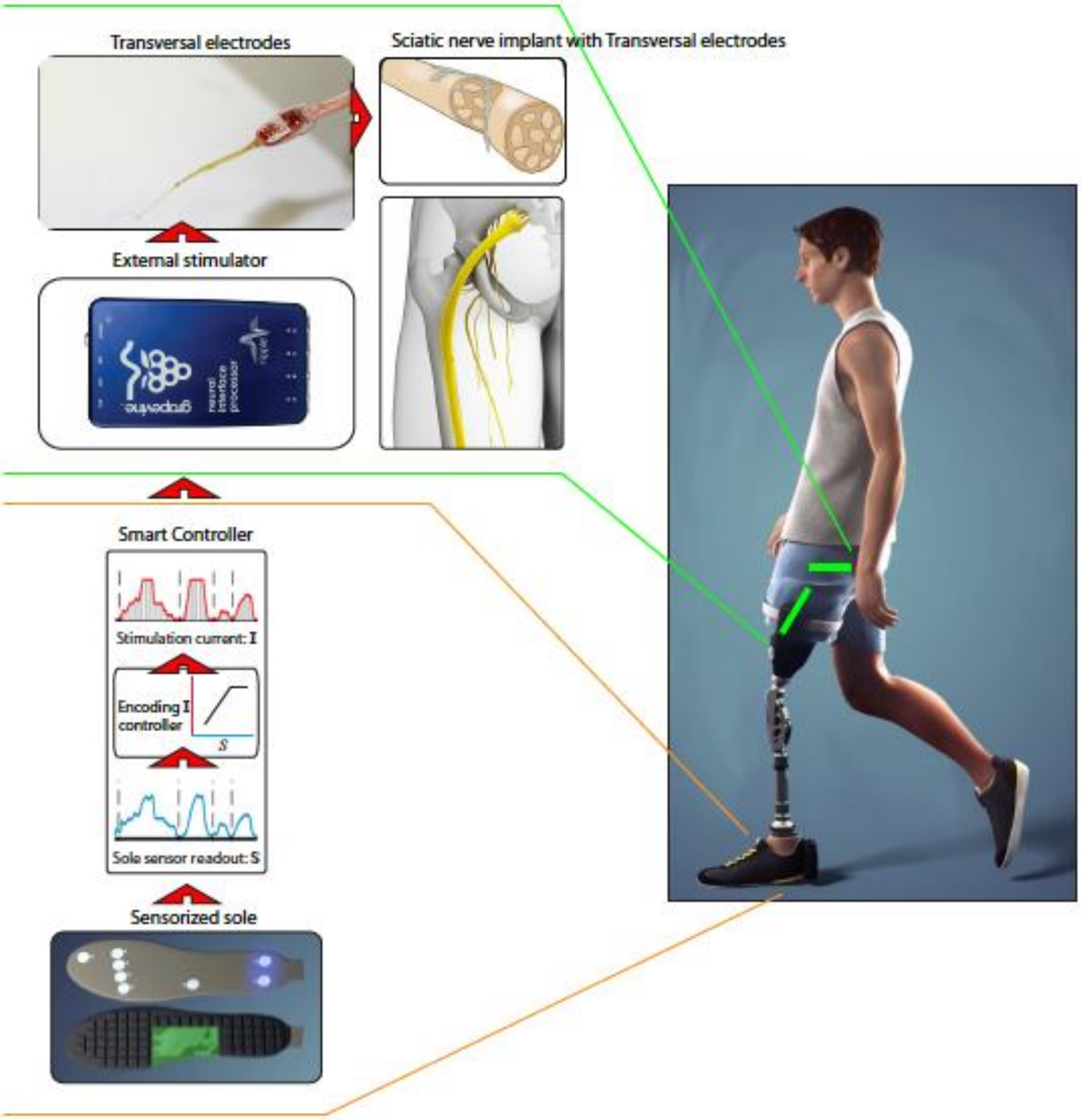

Figure 1: The FeelAgain final demonstrator and its main components

2. SCREENING

A patient fulfilling all inclusion and exclusion criteria will be included in the study and will start the experiment.

Inclusion criteria

- Uni-lateral transfemoral amputation above the knee level
- Other treatments for phantom limb pain should have been tried with poor results
- The subject should experience phantom limb pain at a level of 6 or higher measured on a visual analog scale (VAS) ranging from 0-10
- Phantom limb pain should be experienced at least once a week
- The subject should be in a chronic and stable phase, and the stump should have healed

- The subject should otherwise be healthy and able to carry out the experiment
- If pain medication is used it will be acceptable that the person continues to use the medication
-

If any inclusion criteria are ticked "no" then the patient is not eligible for the study.

Exclusion criteria

- Cognitive impairment
- Pregnancy
- Prior or current psychological diseases such as borderline, schizophrenia, depression or maniodepression
- Acquired brain injury with residual impairment
- Prior neurological or musculoskeletal diseases
- History of or active substance abuse disorder
- Excessive sensitivity to electrical stimulation with surface electrodes
- Persons with fear for electrical stimulation, pain cannot participate
- Persons that are hypersensitive to electrical stimulation and experience the stimulation as unpleasant cannot participate
- Since the protocol includes MRI scanning of the brain, persons that may feel claustrophobic cannot participate

If any exclusion criteria are ticked yes then the patient is not eligible for the study.
During all the experiment the patient included in the study will receive a psychological support if needed. The needs will be decided from the neurologist involved in the trial that will have a close and daily relationship with the patient.

3. THE PROTOCOL

<u>The protocol is divided into different phases that are (in chronological order):</u>

- Baseline(T0);
- Implant(T1);
- Intervention period(T2);
- Explant(T3);
- Follow-up(T4).

Baseline (T0)
During this phase, the phantom limb pain will be recorded.
Details on the methods that will be used to perform these procedures are provided in the paragraph **”Intervention method, Program 2”**.

Implant (T1)
This phase will last one week. Apart the implant of intraneural electrodes, during this phase the phantom limb pain, and the mood of the patients will be recorded.
The detailed surgical procedure of the electrodes implant follows.

Implant of intraneural electrodes

The surgical procedure will be based on the procedure used for the implant of TIME electrodes[26].
The sciatic nerve conveys majority of somatosensory innervation of the foot and ankle, and it is composed of two main components: the tibial and the peroneal one. Thanks to peripheral nerve ultrasound it is possible to visualize the sciatic nerve and its biggest fascicles easily from the gluteal fold. The implant of 2 electrodes will be performed inside the sciatic nerve in an operative room under general anesthesia[26,29]. The line of the incision for electrodes insertion is going to be over the sulcus between the biceps femoris and semitendinosus muscles, in the middle of the posterior aspect of the thigh, starting 4 to 5 cm proximally to the end of the amputation stump. The posterior femoral cutaneous nerve will be identified and protected in order to avoid possible neuropathic pain. Moving semitendinosus medially and biceps femoris laterally the sciatic nerve will be identified.
Once the electrode cables have been tunneled through counter-incision, the rectangular connector of the electrode (inter-connect block) will be placed along the nerve, anchoring it to the surrounding connective tissue with non-absorbable 4-0 silk threads, so as to prevent that twisting forces applied to the electrode cables can move the electrode. With the aid of the intraoperative microscope, the surgeon will plan the entry point of the electrode in the nerve, and will make a minimal external neurolysis enough to see "in transparency" the nerve fascicles.
A guiding needle and suture will be used to insert the electrode in the nerve and to place the active sites inside appropriate nerve fascicles. The guiding needle, which will be used, is a straight needle (125 µm diameter, 26°tipangle) with a pre-attached loop suture (10-0; EthiconEH7900).
The needle will be passed across some of the nerve fascicles, and then used to slowly pull the electrode inside the nerve until the active sites of the electrode will be placed in the correct position, i.e. inside nerve fascicles. These maneuvers will be carried out with great caution to avoid the filament suture breaking at the connection point with the electrode thread. Thanks to the surgical microscope, the surgeon will be able to check the correct positioning of the electrodes active sites inside the nerve. At this point the surgeon will cut the suture of connection and withdraw the needle-suture.

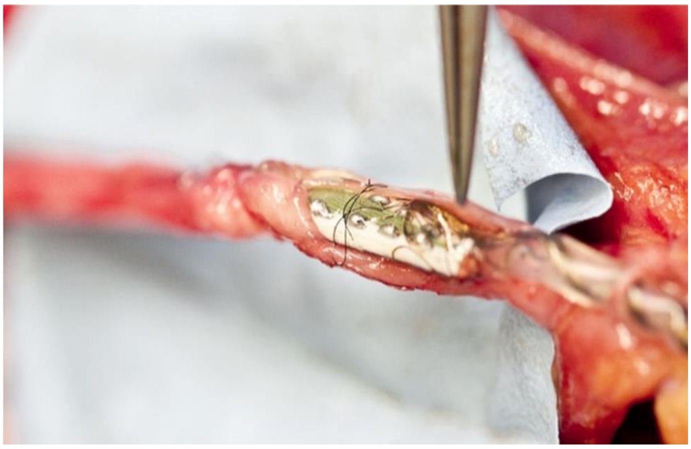

Figure 2. Picture showing the first possible way proposed to anchor the ceramic adapter to the nerve.

The electrode ribbon and the ceramic connector will be anchored to the nerve as shown in the Figure 1 and Figure 2, as this would not induce any compression and it would avoid any

problem for the interface of the electrode embedded into the connective tissue of detaching from the nerve. The type of anchoring will be chosen according to the surgeon preference considering the anatomical situation.
As soon as the electrode is implanted it will be connected with the stimulator and an isolated measurement system, in order to check the correct positioning through the impedances analysis, thus identifying the well-functioning active sites. The aim of this intra-operative session will be to understand if the electrodes are well inserted in the nerves and if are not damaged and can reliably provide stimulation of sensory fibres. Indeed, the current generated by the stimulator through the implanted electrodes will be measured and checked together with the voltages seen on contacts of the electrodes.
At this point the three anchorage holes of the electrodes will be sutured to the epineurium through non-resorbable 8-0 suture.
The flexible electrode cables for connection will be housed with one or more loops in the previously prepared subcutaneous pockets in order to reduce the tensile forces on the electrode. The loops will be anchored to the muscle and facial tissue with 4-0 silk wire suture.

Figure 3. Picture showing the second possible way proposed to anchor the ceramic adapter

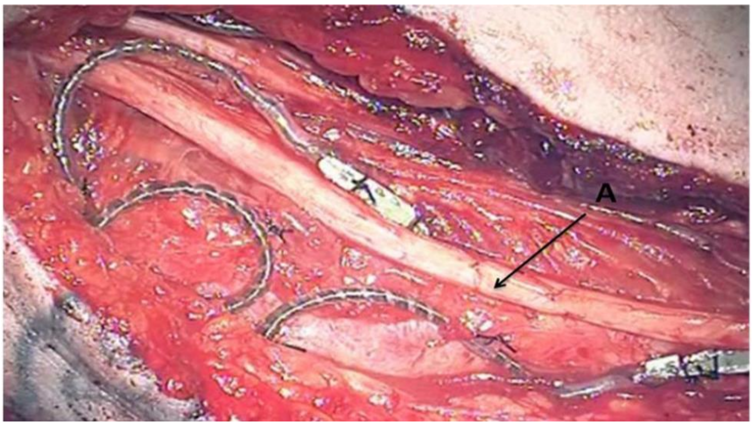

to the nerve.

The external part of the leads will be finally anchored with a suture point to the skin surface performing one or two loop in order to reduce the tensile forces on the cable. This will enable the electrodes to then be subsequently connected with the stimulator.
The procedure will then be repeated for the other electrode taking care to avoid intersections between the electrodes and the output cables. Each electrode will have an external marking with different colors to ensure easy identification in the post-surgery.
The surgery for electrode implanting will probably take several hours (at least 4 hrs). The surgeons that will perform the study were trained for this specific procedure on human cadavers and animal models.

Perioperative prophylactic antibiotics:
Cefuroxim – 1.5 gr (or similar antibiotics) starting ½ hour before surgery and repeated every 4 hour the day of the surgery.

Postoperative prophylaxis:
Routinely use of prolonged prophylaxis will be not performed due to risk of promoting growth of resistant strains of bacteria.

Daily wound-care will be performed with application of Chlorhexidine 0.5%.

Intervention period (T2)
Few days after the surgery – after inspection and disinfection of the surgical wounds – the subjects will start daily sessions of nerve stimulation. Programs of stimulation (Pr) will be performed every day (except on weekends) for up to 6 hours per day according to the daily experiment aims and the compliance of the patient.
The trial will be divided into two different phase:

1. intensive (from 1st to 6th week); during this period the experiments will be carried out every day, from Monday to Friday;
2. semi-intensive (from 7th to 12th week); during this period the experiments will be carried out only three days per week, that patient would prefer;

The stimulation protocol will be composed of different programs, each one with a different aim. The different parts of the experiment will be not necessarily separated from one another; for example two different Programs could be performed on the same day, according to the daily experiment aims.
Program 1
The **program 1** (Pr1) of the experiment will be performed in order to characterize each channel of the electrodes and specifically with the aim of identifying:

- The location, type and strength of the generated sensation with respect to the active sites used to generate them**;**
- The lower (thresholds) and upper (saturation) limits of the current to be delivered in order to induce meaningful sensations (defined respectively as the lowest stimulus pulse charge at which the subject could reliably feel a sensation and the pulse charge at which the sensation becomes close to uncomfortable or painful);
- Electrode channels, that are useful and able to induce reliable sensory responses in the patients phantom foot or leg (the sensation will be considered reliable if when using the same characteristics of current, the generated sensation will be the same considering the strength, the location and the type in at least two out of three repeated trials of stimulation; the charge delivered for the three repeated trials will be between the lower thresholds and upper limits).

For this purpose each channel of each electrode will be connected with the electrical stimulator. The activation of the stimulator will be able to deliver short trains of electrical current of variable intensity, duration, frequency and shape. The stimulator - and consequently the parameters of the current to be injected into the nerve - will be managed by the operators (the team of clinicians and biomedical engineers) through a dedicated platform, which is a low level and embedded software specifically designed for the purpose.
The charge applied must never exceed the maximum value that produces irreversible electrochemical reactions at the active sites of the electrode. The electrical stimulator embeds a safety procedure that monitors the charge injected and stops stimulation if it exceeds the maximum authorized one.
The parameters of stimulations will be set on the base of previous sensory stimulation experiments[29-30]. More in particular the pulse length will be in the range from the minimum of 1 microsecond to 10 milliseconds and varied with a minimal resolution of 1 microsecond (and commonly in multiples of it); the intensity in the range from 10 µA to 0.95 mA with resolution of at minimum 7.5 µA (and will be varied most commonly in multiples of 10 µA); the frequency in the range from 1 Hz to 1 KHz with a resolution of 1 Hz (and varied in multiples of it). The mapping will be performed by means of an algorithm similar to the one showed in Figure 5 (that is a non-limiting example of the intended use).

During the mapping procedure first one electrical parameter will be modulated and two others will be maintained fixed (e.g. varying intensity (I) while maintaining fixed the pulse length (PL) and the frequency (F)). Then the same will be done with the second parameter, and finally third, finding the range of parameters (I, PL, F), which elicit meaningful sensations. The single ranges for all parameters will be determined with respect to the measured impedances, and precedent experimental experiences, and will be made ad-hoc for every individual involved. Then eventually, the time occurrence and amplitude of single pulses will be simultaneously modulated.
The order of modulation for the single parameters will be randomized, so to bias the user, and assure that there will not occur the habituation.
The patients will provide his/her feedback by means of a dedicated psychophysical platform specially designed for this purpose.
An example of train of stimuli is reported: 500ms trains of charge–balanced, biphasic, rectangular pulses will be delivered 300 μs duration, frequency of 90 Hz and amplitude increasing from 20 to 140 μA (with steps of 20μA).

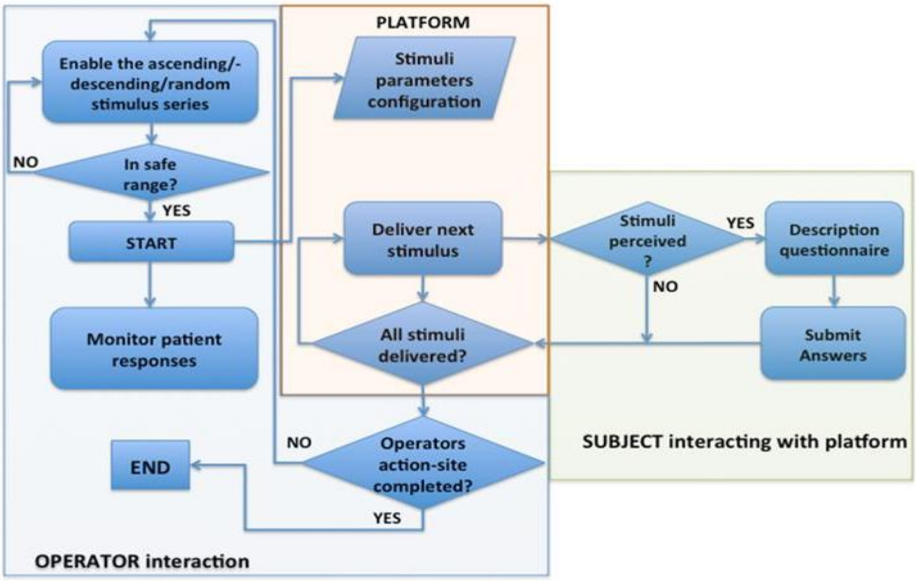

Figure 5. Proposed flowchart for the mapping procedure (this is an example, of several, possible intended use modes)

The procedure will be similar (but not limited to) as follows: the first low amplitude stimulus (i.e. 20 μA in the case of the above mentioned example) will be delivered; if no sensation will be felt in the phantom foot by the patient following the first stimulus, the next stimulus will be delivered (i.e. 40 μA in the case of the above mentioned example), and so on, until the patient reports a mild sensation. This is when he will first interact with the platform and describe details of the sensation perceived. After this we will continue to deliver increasing stimuli and we will continue to record the feedback from the patients until the generated sensation felt becomes uncomfortable.
The same procedure will be repeated again twice with the same characteristics. If the same sensation is reported at least once, the first pattern of electrical parameters, which is able to generate the mild sensation, will be identified as the lower charge threshold and the one able to generate an uncomfortable sensation (not still defined by the patient as pain) will be identified as the upper limit. These electrical parameters will be recorded together with the type, the localization and the intensity of the sensation reported by the patients with a dedicated interface.

The time required to carry out this experiment will be about 320 minutes (cables connections setup included), divided between morning and afternoon.
This part of the protocol will be performed:

- during the intensive phase:
  - the first week after the implant;
  - the first day the following weeks (in order to record possible changes of the location, type and strength of the generated sensation and of the lower threshold and upper limits of the charge, over the weeks).
- during the semi-intensive phase:
  - the first day of the weeks (in order to record possible changes of the location, type and strength of the generated sensation and of the lower threshold and upper limits of the charge, over the weeks);

Program 2
Program 2 (Pr2) will be aimed to challenge the walking ability of the patients with the prosthesis with sensory feedback. During the Pr2 we will assess the effectiveness of SENSY through a battery of tests:

Fall avoidance and walking preservation during perturbed standing and locomotion
During the fall avoidance and walking preservation test, the subjects are asked to walk at their self-determined maximum comfortable speed, through a platform endowed with randomly disposed climbing grips of different sizes (from 3x3x1 cm, to 7x7x7 cm). The platform is created for fitting in parallel bars (5.50 x 0.56 m), which will be used as supports in case of falls of the participants. Subjects will be asked to not rely on the bars during walking. During the task, they will wear modified glasses which will prevented them from watching their steps and therefore from knowing where the obstacles were placed. Errors will be counted when the subjects, after stepping on an obstacle, lose balance, and had to rely on the bars (either by grasping them or posing the body over them) to prevent a fall. The error count could be computed as the ratio between falls and number of total trampled obstacles in the session.
Among possible analysis that could be performed: offline videos inspection by operators, center of pressure (CoP)[31] position recorded by a force transducers and lateral Center of Mass (CoM) and extrapolated center of mass (XcoM)[32] position could be computed by filtering the CoP data.

Gait analysis
Subjects will walk over ground indoor and outdoor and on a treadmill, in a wide range of speeds, while the trajectory of a set of markers and sensors located on suitable body landmarks will be recorded by the motion capture system. Temporal and spatial kinematic reconstruction (velocity (m/min), cadence (steps/min), stride length (m), reported confidence, among the others[1-10], ground reaction force (vertical ground reaction force) will be used to estimate the degree of asymmetry induced by the prosthesis. The same protocol will be used during the following tasks: climbing and descending stairs; climbing and descending ramps; initiating and terminating walking; sit-to stand transition; turning; straight-line walking test. These could be performed in normal and dual task[33] modality.

Metabolic cost will be periodically evaluated by means of standardized tests (e.g. Cardiopulmonary exercise testing (CPET): rate of oxygen uptake, net oxygen cost, maximum aerobic capacity, relative energy cost and heart rate[10]), under different operative conditions.

Phantom Limb Pain

According to the Initiative on Methods, Measurement and Pain Assessment in Clinical Trials (IMMPACT) guidelines[34-35], it is advisable to include different chronic pain outcome domains to evaluate the intensity and duration of pain, and its influences on quality of life.

Clinical rating tests to perform in the clinical trials include:

- Map of the pain perception (MAP-pain)

Specific aim of test: Localization and tracking of perceived pain.

Protocol for data collection: the areas of foot/leg where the subject feels phantom pain will be marked in a drawing of the lower extremity. Different shadows will be used to identify different intensities of the pain. Data description/interpretation: drawing outlining the areas of the lower extremity where the subject feels the phantom limb pain topographically annotated by the intensity of the pain.

- Numerical rating scale (NRS)

Specific aim of test: subjective quantification of pain

Protocol for data collection: it is accompanied by the instruction “please rate your pain by indicating the number that best describes your pain on average in the last 24 hours”. A visual-analog scale (VAS) will be provided to facilitate scoring.

Data description/interpretation: a range of 0-10, where a score of 0 equals ‘no pain’ and 10 equals ‘pain as bad as you can imagine’.

- The Neuropathic Pain Symptom Inventory (NPSI)

Specific aim of test: evaluates the different symptoms of neuropathic pain; the NPSI allows discrimination and quantification of the distinct and clinically relevant dimensions of neuropathic pain syndromes (spontaneous on-going pain, spontaneous paroxysmal pain, evoked pain, and paraesthesia/dysaesthesia).

Data collection: the subject is asked to respond to a 12 question inventory rating the average pain severity during the past 24 hours.

Data description/interpretation: The final version of NPSI includes 12 items: 10 are descriptors of the different symptoms and 2 assess the duration of pain. A total intensity score is calculated as the sum of the scores of the 10 descriptors.

Rubber Foot Illusion (RFI).

Amputees will look (through head mounted display, HMD) at a virtual replacement of their foot that will be periodically illuminated. The illumination will match the spatial location of the neural-stimulation-elicited tactile stimulation and will flash either synchronously with it, or with a fixed delay. The sense of foot ownership will be measured by asking participants to rate on a visual analogous scale their agreement to a number of statements assessing how much they felt the virtual foot to be their own foot. As an implicit measure of foot ownership, we will also measure the perceived position of participants’ own foot (proprioceptive drift: perceived position of the real foot should “drift” towards that of the virtual foot).

The same experiment can be alternatively performed without HMD, where light stimuli will be changed with touch from an experimenter.

Cognitive load:

EEG data acquisition will be performed with a sampling frequency of 500 Hz and 24-bit data resolution. The small EEG amplifier will be tightly connected to a 24-channel sintered Ag/AgCl electrodes cap, (Easycap, Germany) at the occipital site of the participants’ head, using an elastic

band. Electrodes will be placed according to the international 10-20 System. The electrodes will be referenced to FCz and the ground electrode will be AFz.
A three-class oddball auditory task[36] will be used for evaluating a dual task performance, through investigation of the P300 event-related potential (ERP) component's amplitude, as proposed by[37-38]. A standard tone and two deviant tones will be delivered to the participants in a random order through commercial headphones. The participants' task will be to silently count the target tones and ignore the standard and deviant tones, while in sitting (control condition) or walking on hard surface when the nerve stimulation will be switched off, or on. The walking route will be predefined and practiced before the start of the experiment. The order of the conditions will be balanced and randomized between subjects. Every condition will be repeated twice. The travelled distance in both conditions will be similar for both subjects. The grand average ERPs across participants will be computed for both the "Target" and "Non-target" conditions.

Functional Magnetic Resonance brain imaging.
Subjects will run 2 sessions of functional MR imaging of the brain to assess eventual effect of the treatment on the plasticity and the connectivity of the brain. The sessions will be scheduled immediately after explant and several months later as a follow-up.

Quality of life questionnaires
The quality of life of patients will be assessed by means of The Patient Health Questionnaire (PHQ-9) and with self-reporting about eventual benefits and defects induced by the treatment.
PHQ consists in 9 questions that are completed by the patients and scored by the clinician. The questions incorporate DSM-IV depression diagnostic criteria with other major depressive symptoms. PHQ-9 scores of 5, 10, 15 and 20 represent mild, moderate, moderately severe and severe depression.

The Pr2 will start the fifth week after the implant and will be afterwards repeated every day of the trials. Pr2 will last about 1.5 hours per day according to patients' availability.

Explant (T3)

The explant procedure will be executed 3 months after the implant, according to the desire of the patient, or if the malfunctioning of the system is observed. This phase will last one week (surgery and hospital stay). In the operating room, under a general anesthesia, the patient will be placed in the same position as during the course of the implant operation. After the removal of the anchor suture point of the connection cable to the skin, the surgeon will reopen the previous incision and will expose the sciatic nerve. The connection cable and the electrode inside the patient's leg. After the positioning of a silicone cap to preserve the connector, the surgeon will close the wound with non-absorbable sutures 3-0. Therefore the system could be ready for the future use with the fully implantable connector.
Instead, in the case of the electrodes removal, the connection cable and the electrodes will be dissected from scar tissue using microsurgical technique, the stitches inserted during the implant procedure will be removed and the electrodes and connection cables carefully pulled out. At the end of this procedure the surgeon will go on to close the wound with non-absorbable sutures 3-0. The part of neural tissue, of the amputated nerve, distal to electrodes and in electrode section will be dissected, avoiding complications or neuroma formation to the patient. These will be also sent for the future histological analysis, prior the patient's acceptance in the informed consent.

In case of an unlikely infection the electrodes and connection cables will have to be removed at the end of the trial or before planned if antibiotics treatment will be not able to treat the pathology. The system will be also removed in case of other important adverse event related to the implanted system (as bleeding or nerve damage) not treatable conservatively.

Follow-up (T4)

During the follow-up phase, pain measures will be executed right after the explant, and up to the three months after the explant. Also the MRI measurements will be performed.

4. RISKS AND BENEFITS

A detailed analysis of risk and benefit is included in the overall project. Moreover a detailed Risk analysis was performed for each of the medical devices that will be used during the project. A Risk Management paragraph is included in the Manual User of the different devices.

Monitoring of results
Results will be collected by using a dedicated software called Psychophysical Platform. More detail about this software can be detected on the Investigator Brochure. Moreover a paper CRF will be used.

Potential Risks related to the Study Procedures
The implant of the intraneural electrodes is a surgical procedure performed with a single skin incision of approximately 10 cm to expose the nerves to be implanted. This procedure exposes to the general risks associated with any surgery, including mortality risk, that are:
- bleeding disorders (as post-surgical hematoma formations that can compress the nerves of the amputee upper limb),
- infections,
- local lesion of lymphatic system in the amputee limb with a consequent risk of chronic swelling and tenderness of the stump,
- vessels lesions of the amputee limb.

These complications may require an extension of amputation toward the hip, or alterations modifying the shape of the stump and/or the possibility of residual movements that could make difficult in the future the use of prostheses.
The intervention of implanting electrodes inside the nerves is an experimental procedure that could be associated with specific risks of injury and/or degeneration of the nerves of the stump which can lead to an impairment or loss of motor function, sensitivity and proprioception of the distal stump than the previous condition. Moreover foreign body reaction, postsurgical fibrous scar (scar) around the nerves, and the formation of local neuromas (painful swelling) could be possible.
Despite the implanted electrodes are made of a biocompatible material, there may be an intolerance of the same, rejection or non-functioning of the electrodes, with the needs of a surgical revision to remove them before the planned time.
The electrodes implanted are soldered to wires for the percutaneous connection with the external stimulator. This exposes to an increased risk of local complications such as infections, painful scars, skin rashes, and the possibility of injury from stripping (even accidentally) in the postoperative period with consequent nerve injury where the electrodes are applied.

All these conditions may require medical or surgical treatment and cannot excluded also the possibility of residual lesions that can prevent a further implantation of intraneural electrodes to the same nerves in the future.
The surgical electrodes removal at the end of the project can determine the same complications above reported for the implant intervention.
The electrodes are made of biocompatible materials. The long-term use of such electrodes has already been tested in a EU study called EPIONE, already approved by the Italian, Swiss and Danish Ministries of Health (Department for medical devices) and currently underway. For this reasons there are not expected side effects due to long-term maintenance of the electrodes (or part of its) inside the nerve in a patient.
An excessive stimulation, in terms of injected charge, could cause pain (even intense) or a nerve damage with the risks of injury and/or degeneration of the implanted nerves leading to an impairment or loss of motor, sensory and proprioceptive function of the distal stump.

Risk Minimization Actions Throughout the Protocol
In case of infection the electrodes and connection cables could be removed before the end of the trial if antibiotics treatment is not able to treat the pathology.
A surgical revision of the system could be performed and eventually the electrodes could be also removed before planned in case of other important adverse events, as bleeding or nerve damage, related to the implanted system not treatable conservatively.
The stimulators include a safety system controlling that the charge of injected current does not exceed a level that could damage the electrodes and the nerves.
A careful risk analysis with related risk management was performed for each of the used medical devices.

Benefit
A decrease of Phantom Limb Pain, if present in the recruited patient, could be expected.
During the trial the patient will be trained to the use of the prosthesis with sensory feedback and, in the case of future commercialization of a similar system, will be considered for a use of the system in case of successful use.
Data obtained with the trial will be used in order to improve the knowledge on the use of leg prosthesis with sensory feedback on amputee patients.

5. AMENDMENT

According to the UNI EN ISO 14155 (2012) all the proposed substantial amendment (concerning the safety, the scientific validity and management of the study and patient) will be submitted by the Promoter to the Ethical Committee before being applied.
According to the Promoter's decision no substantial amendment will be communicated to the Ethical Committee.

6. EFFICACY ENDPOINT

Impact of SENSY on mobility, on falls avoidance, on metabolic consumption

<u>Secondary endpoints:</u>

- Impact of SENSY on phantom pain
- Impact of SENSY on embodiment
- Impact of SENSY on cognitive effort
- Impact of SENSY on cortical plasticity

Endpoint Security
Safety will be monitored during the whole study concerning the incidence, nature and severity of any adverse events related to the use of the prosthetic system. Any adverse events will be promptly communicated to the Ethics Committee of Clinical Center of Serbia in Belgrade. The definition of adverse events is in agreement with MEDDEV 2.7.3.

7. TRAINING

The surgeon has previous experiences in the implant/explant of intraneural electrodes on cadavers. The team of biomedical engineers that will participate to the study has already experience with clinical trial with implant of intraneural electrodes and devices for restoring sensory feedback.

8. DATA PUBLICATION

The data obtained with the experiment will be published, in agreement with the whole consortium, in peer-reviewed international journals with IF.

9. INFORMED CONSENT

A written informed consent will be obtained from the patient before being recruited in the study.

10. REFERENCES

1. Tucker, M. et al. *J Neuro Eng Rehab***12**,1 (2015).
2. Miller, W. et al. *Arch Phys Med Rehabil***82**(8):1031-7. (2001)
3. Nolan, L. et al. *Gait Posture***17**, 142–151 (2003).
4. Miller, W. et al. *Phys Therapy***82**, 856–865 (2002).
5. Hof, A. et al. *Gait Posture***25**, 250–258 (2007).
6. Vrieling, A. et al. *Gait Posture***27**, 423–430 (2008).
7. Vrieling, A. et al. *Gait Posture***27**, 82–90 (2008).
8. Burke, M. et al. *Ann. Rheumatic Diseases***37**, 252–254 (1978).
9. Gailey, R. et al. *J Rehab Res Dev***45**, 15 (2008).
10. Waters, R. et al. *J Bone Joint Surg Am***58**, 42-6 (1976).
11. Czerniecki, J. et al. *Disability and rehabilitation* 1-9 (2015).
12. Flor, H. et al. *Nat Rev Neurosci* **7**, 873-81 (2006).
13. Flor, H. et al. *Lancet Neurol***1,** 182-9 (2002).
14. Williamson G. et al. J. *Social Behavior Personality* **9**, 249–268 (1994).
15. Heller, B. et al. *Clin Rehabil***14**, 518–522 (2000).
16. Blanke, O. et al. *Nat Rev Neuroscience***13**, 556-571 (2012).

17. Botvinick, M. et al. *Nature* **391**,756-756 (1998).
18. Gailey, R. et al. *J Rehabil Res Dev***47**, 317-32 (2010).
19. Fleury, A. et al. *Geriatrics & gerontology international***13**, 264-273 (2013).
20. Roll, R. et al. *Neuroreport***13,** 1957-61 (2002).
21. Kennedy, P. et al *J Physiol* **538**, 995–1002 (2002).
22. Trulsson, M. et al. *Exp Brain Res* **137**,111-6 (2001).
23. Nurse, M. et al. *Clin Biomech***16**, 719–727 (2001).
24. Perry, S. et al. *Brain Res***877,** 401-6 (2000).
25. Boretius, T. et al. *Biosens. Bioelectron*. **26**, 62–69 (2010).
26. Raspopovic, S. et al.*Sci Transl Med* **6**, 222ra19 (2014).
27. Badia, J. et al. *J. Neural Eng***8,** 036023 (2011).
28. Dominici, N. et al.*Science***334**, 997-999 (2011).
29. Rossini, P., et al. *Clin Neurophysiol*. **121** 777-3 (2010).
30. Raspopovic, S. et al. *IEEE Trans Neural Syst Rehabil Eng***19**, 333-344 (2011).
31. Pai, Y. et al. *J Biomech***30,** 347-54 (1997).
32. Hof, A. et al. *Hum Mov Sci***27**, 112-25 (2008).
33. C. Wickenset al , *Science* **221**, 1080–1082 (1983).
34. Dworkin RH, et al. J Pain Off J Am Pain Soc. 2008Feb;9(2):105–21.
35. Dworkin RH, et al. Pain. 2012 Jun;153(6):1148–58.
36. R. Zink, et al, *J Neural Eng* **13**, 046017 (2016).
37. J. Polich, et al, *Clinical neurophysiology* **118**, 2128–2148 (2007).
38. T. W. Picton, et al, *Journal of clinical neurophysiology* **9**, 456–479 (1992).

2. STATISTICAL ANALYSIS

1. DESCRIPTION OF THE STATISTICAL METHODS

After monitoring, data will be acquired by different partners of the study (e.g. Clinical Center of Serbia, SensArs Neuroprosthetics, Swiss Institute of Technology Lausanne, Switzerland) to guarantee the integrity of the data itself in accordance with the principles of the Guide to Good Clinical Practices of the European Community.
The statistical analysis will be carried out at both the Swiss Institute of Technology Lausanne and SensArs Neuroprosthetics.
All statistical tests will be two-sided, with p-values of 0.05 or less denoting statistical significance. Parametric or nonparametric tests will be used after verifying whether the distributions of data are estimated by a gaussian distribution or not.

2. PROPOSED NUMBER OF PERSONS TO BE INCLUDED IN RESEARCH

This study is conceived as a proof-of-concept to assess whether there is an impact of sensory feedback restored through intraneural stimulation on leg amputees' use of the prosthesis and on phantom limb pain. Because of this reason, we plan to enroll 3 subjects. No planning has been done

for determining the sample size for this study. Instead, planning has been done for determining the numerosity of the measurements in the tasks to assess the impact of direct nerve stimulation on amputees' health.
In particular:

- Gait analysis tests are designed after standard tests[1]
- Cognitive burden assessment is designed reproducing the dual task proposed by Zink et al.[2]
- Metabolic consumption is based on[1,3,4]
- Pain therapy is designed after the protocol of Soin and colleagues[5]: the patients will receive a session of therapy when they report an attack of pain. A minimum of 10 therapies (plus controls) will be delivered.

3. METHOD OF TAKING INTO ACCOUNT MISSING, UNUSED OR UNAUTHORIZED DATA

Any patient that will execute all the tasks until follow-up will be included in the analysis.

4. MANAGEMENT OF AMENDMENTS TO THE INITIAL STRATEGY ANALYSIS PLAN

Any significant changes to the analysis scheme will be submitted to the ethics committee for approval as an amendment to the study.

5. REFERENCES

1. Steffen, TM et al. *Physical therapy* **82**: 128–137 (2002).
2. Zink R, et al. *Journal of Neural Engineering* **13**: 046017 (2016).
3. Waters RL, et al. *J Bone Joint Surg Am* **58**: 42–46 (1976).
4. Traballesi M, et al. *Gait & posture* **27**: 70–75 (2008).
5. Soin A, et al. *Neuromodulation: Technology at the Neural Interface* **18**: 197–206 (2015).
