# Supplementary material for: Biomimetic computer-to-brain communication enhancing naturalistic touch sensations via peripheral nerve stimulation
Source: Nat Commun. 2024 Feb 20;15:1151. doi: 10.1038/s41467-024-45190-6 (PMC10879152; doi:10.1038/s41467-024-45190-6)
Supplement: Supplementary file 3 — Reporting Summary [file 41467_2024_45190_MOESM3_ESM.pdf]

nature portfolio

Corresponding author(s): Stanisa Raspopovic

Last updated by author(s): Dec 22, 2023

# Reporting Summary

Nature Portfolio wishes to improve the reproducibility of the work that we publish. This form provides structure for consistency and transparency in reporting. For further information on Nature Portfolio policies, see our Editorial Policies and the Editorial Policy Checklist.

## Statistics

For all statistical analyses, confirm that the following items are present in the figure legend, table legend, main text, or Methods section.

| n/a | Confirmed | |
|---|---|---|
| ☐ | ☒ | The exact sample size (*n*) for each experimental group/condition, given as a discrete number and unit of measurement |
| ☐ | ☒ | A statement on whether measurements were taken from distinct samples or whether the same sample was measured repeatedly |
| ☐ | ☒ | The statistical test(s) used AND whether they are one- or two-sided<br>*Only common tests should be described solely by name; describe more complex techniques in the Methods section.* |
| ☐ | ☒ | A description of all covariates tested |
| ☐ | ☒ | A description of any assumptions or corrections, such as tests of normality and adjustment for multiple comparisons |
| ☐ | ☒ | A full description of the statistical parameters including central tendency (e.g. means) or other basic estimates (e.g. regression coefficient) AND variation (e.g. standard deviation) or associated estimates of uncertainty (e.g. confidence intervals) |
| ☐ | ☒ | For null hypothesis testing, the test statistic (e.g. *F*, *t*, *r*) with confidence intervals, effect sizes, degrees of freedom and *P* value noted<br>*Give P values as exact values whenever suitable.* |
| ☒ | ☐ | For Bayesian analysis, information on the choice of priors and Markov chain Monte Carlo settings |
| ☒ | ☐ | For hierarchical and complex designs, identification of the appropriate level for tests and full reporting of outcomes |
| ☐ | ☒ | Estimates of effect sizes (e.g. Cohen's *d*, Pearson's *r*), indicating how they were calculated |

*Our web collection on statistics for biologists contains articles on many of the points above.*

## Software and code

Policy information about availability of computer code

| | |
|---|---|
| Data collection | Data from the sensorized insoles were collected through a custom software made in C++ running on linux; sensation characterization and stimulation routines adopted in the animal experimentation were implemented and data saved through a custom-made software written in Matlab. For animal experiments, we provided the stimulation using AM stimulators Model 2100 (A-M Systems, Sequim, WA, USA). Electromyographic and neural signals were acquired using the LTR-EU-16 recording system with LTR11 ADC (L-Card, Moscow, Russia) and the RHS recording system with 32-channel headstages (Intan Technologies, Los Angeles, CA, U.S.A.) at a sampling frequency of 25 and 30 kHz respectively. |
| Data analysis | Data were analyzed in Matlab (R2020a, The MathWorks, Natick, USA) and Python (3.7.3, the Python Software Foundation), using “SciPy” and “NumPy” packages |

For manuscripts utilizing custom algorithms or software that are central to the research but not yet described in published literature, software must be made available to editors and reviewers. We strongly encourage code deposition in a community repository (e.g. GitHub). See the Nature Portfolio guidelines for submitting code & software for further information.

## Data

Policy information about availability of data

All manuscripts must include a data availability statement. This statement should provide the following information, where applicable:

- Accession codes, unique identifiers, or web links for publicly available datasets
- A description of any restrictions on data availability
- For clinical datasets or third party data, please ensure that the statement adheres to our policy

The neural data recoded during the animal experiments, and data from the human experiments that support the study findings are provided at https://github.com/NatalijaKatic/Biomimetic-project.git. Protocol for human clinical trial is given as part of the Reporting Summary. Any additional explanation of datasets, or data presented in another form, are available by request from the corresponding author. Source data are provided with this paper.

## Research involving human participants, their data, or biological material

Policy information about studies with human participants or human data. See also policy information about sex, gender (identity/presentation), and sexual orientation and race, ethnicity and racism.

| | |
|---|---|
| Reporting on sex and gender | The three participants recruited for this pilot study were all males, identified as males |
| Reporting on race, ethnicity, or other socially relevant groupings | The three participants recruited for this pilot study were all White |
| Population characteristics | Transfemoral (thigh-level) amputees proficient in the use of commercial prosthesis and affected by phantom pain. All of them were active users of passive prosthetic devices (Ottobock 3R80). Table S1. shows Participants' demographics. |
| Recruitment | The participants were recruited among the population of transfemoral amputees who executed rehabilitation at the prosthetic center at the Clinical Center of Belgrade. The participants were interviewed by doctors to assess eventual mental impairment and eventual other exclusion criteria (see protocol). All the participants read and signed the informed consent. We carefully considered the study design and the questions being asked during the recruitment process to minimize potential sources of self-selected bias. The patients received reimbursement covering all the expenses during the clinical trial. |
| Ethics oversight | Ethical approval was obtained from the institutional ethics committees of the Clinical Center of Serbia, Belgrade, Serbia, (ClinicalTrials.gov identifier NCT03350061). |

Note that full information on the approval of the study protocol must also be provided in the manuscript.

# Field-specific reporting

Please select the one below that is the best fit for your research. If you are not sure, read the appropriate sections before making your selection.

☒ Life sciences ☐ Behavioural & social sciences ☐ Ecological, evolutionary & environmental sciences

For a reference copy of the document with all sections, see nature.com/documents/nr-reporting-summary-flat.pdf

# Life sciences study design

All studies must disclose on these points even when the disclosure is negative.

| | |
|---|---|
| Sample size | No statistical methods were used to predetermine sample size, because this was a proof of concept trial. |
| Data exclusions | Three participants underwent the surgeries, but, due to work occupancy, one subject decided to participate in a subset of experiments. All the data from the other participants were not excluded. |
| Replication | All the experiments were repeated several times in different days guaranteeing the reliability of results. The numerosity of repetitions for each single experiment is indicated in the manuscript. All attempts at replication were successful. |
| Randomization | The participants run all the same tests in all the same conditions. We compared the participants using or not the biomimetic sensory feedback restoration neuroprosthesis which is the object of this paper. |
| Blinding | The investigators were not blinded to allocation during experiments and outcome assessment. Given the invasive nature of the system, the participants are usually aware of small changes to the system, preventing us from making changes without their knowledge. In all of the experiments, the validity of the results are not biased by the participants' knowledge. |

# Reporting for specific materials, systems and methods

We require information from authors about some types of materials, experimental systems and methods used in many studies. Here, indicate whether each material, system or method listed is relevant to your study. If you are not sure if a list item applies to your research, read the appropriate section before selecting a response.

## Materials & experimental systems

| n/a | Involved in the study |
|---|---|
| ☒ | ☐ Antibodies |
| ☒ | ☐ Eukaryotic cell lines |
| ☒ | ☐ Palaeontology and archaeology |
| ☐ | ☒ Animals and other organisms |
| ☐ | ☒ Clinical data |
| ☒ | ☐ Dual use research of concern |
| ☒ | ☐ Plants |

## Methods

| n/a | Involved in the study |
|---|---|
| ☒ | ☐ ChIP-seq |
| ☒ | ☐ Flow cytometry |
| ☒ | ☐ MRI-based neuroimaging |

# Animals and other research organisms

Policy information about studies involving animals; ARRIVE guidelines recommended for reporting animal research, and Sex and Gender in Research

| | |
|---|---|
| Laboratory animals | Experiments were carried out on 2 adult cats , 2-3 years old, weighing 2.5-4.0 kg |
| Wild animals | The study did not involve wild animals |
| Reporting on sex | Experiments were carried out on 2 adult cats of either sex |
| Field-collected samples | During the experiment, the rectal temperature and mean blood pressure of the animals were continuously monitored and kept at 37 ± 0.5°C and above 80 mmHg. The study did not involve samples collected from the field |
| Ethics oversight | All procedures were conducted in accordance with protocols approved by the Animal Care Committee of the Pavlov Institute of Physiology, St. Petersburg, Russia, and adhered to the European Community Council Directive (2010/63EU). |

Note that full information on the approval of the study protocol must also be provided in the manuscript.

# Clinical data

Policy information about clinical studies

All manuscripts should comply with the ICMJE guidelines for publication of clinical research and a completed CONSORT checklist must be included with all submissions.

**Clinical trial registration**

The study is part of the bigger clinical trial: ClinicalTrials.gov identifier NCT03350061

**Study protocol**

Attached as supplementary material

**Data collection**

The experiments whose results are presented in the paper are part of the bigger clinical trial (ClinicalTrials.gov identifier NCT03350061). The protocol is divided into different phases that are (in chronological order):

• Baseline(T0);
• Implant(T1);
• Intervention period(T2);
• Explant(T3);
• Follow-up(T4).

Baseline (T0)
During this phase, the phantom limb pain will be recorded.
Details on the methods that will be used to perform these procedures are provided in the paragraph "Intervention method, Program 2".
Implant (T1)
This phase will last one week. Apart the implant of intraneural electrodes, during this phase the phantom limb pain, and the mood of the patients will be recorded.
Intervention period (T2)
Few days after the surgery – after inspection and disinfection of the surgical wounds – the participants will start daily sessions of nerve stimulation. Programs of stimulation (Pr) will be performed every day (except on weekends) for up to 6 hours per day according to the daily experiment aims and the compliance of the patient.
The trial will be divided into two different phases:
1. intensive (from 1st to 6th week); during this period the experiments will be carried out every day, from Monday to Friday;
2. semi-intensive (from 7th to 12th week); during this period the experiments will be carried out only three days per week, that patient would prefer;
The stimulation protocol will be composed of different programs, each one with a different aim. The different parts of the experiment will be not necessarily separated from one another; for example two different Programs could be performed on the same

day, according to the daily experiment aims.
Explant (T3)
The explant procedure will be executed 3 months after the implant, according to the desire of the patient, or if the malfunctioning of the system is observed. This phase will last one week (surgery and hospital stay). In the operating room, under a general anesthesia, the patient will be placed in the same position as during the course of the implant operation. After the removal of the anchor suture point of the connection cable to the skin, the surgeon will reopen the previous incision and will expose the sciatic nerve. The connection cable and the electrodes will be dissected from scar tissue using microsurgical technique, the stitches inserted during the implant procedure will be removed and the electrodes and connection cables carefully pulled out. At the end of this procedure the surgeon will go on to close the wound with non-absorbable sutures 3-0. The part of neural tissue, of the amputated nerve, distal to electrodes and in electrode section will be dissected, avoiding complications or neuroma formation to the patient. These will be also sent for the future histological analysis, prior the patient's acceptance in the informed consent. In case of an unlikely infection the electrodes and connection cables will have to be removed at the end of the trial or before planned if antibiotics treatment will be not able to treat the pathology. The system will be also removed in case of other important adverse event related to the implanted system (as bleeding or nerve damage) not treatable conservatively.
Follow-up (T4)
During the follow-up phase, pain measures will be executed right after the explant, and up to the three months after the explant.

The data was systematically collected at the Clinical Center of Serbia, located in Belgrade, Serbia, throughout the years 2017 and 2018.

Outcomes

In this manuscript we were working on the primary endpoint regarding mobility (page 12 of original protocol), and secondary impact on cognitive effort (page 16-17 of original protocol). More in detail, as explained in the added protocol document:

- Mobility-speed (Fig 6a): "Subjects will walk over ground indoor and outdoor and on a treadmill, in a wide range of speeds, while the trajectory of a set of markers and sensors located on suitable body landmarks will be recorded by the motion capture system. (..) The same protocol will be used during the following tasks: climbing and descending stairs; climbing and descending ramps." (page 12 of original IRB approved protocol)

- Cognitive effort (Fig 6b): "These could be performed in normal and dual task modality. "(page 12 of original protocol)

- Naturalness of sensation: "The program 1 (Pr1) of the experiment will be performed in order to characterize each channel of the electrodes and specifically with the aim of identifying: The location, type and strength of the generated sensation with respect to the active sites used to generate them ."(page 10 of original protocol)

## Plants

Seed stocks

N/A

Novel plant genotypes

N/A

Authentication

N/A
